# The segregation of Calb1, Calb2, and Prph neurons reveals distinct and mixed neuronal populations and projections to hair cells in the inner ear and central nuclei

**DOI:** 10.1002/dvdy.70093

**Published:** 2025-10-30

**Authors:** Jeong Han Lee, Ebenezer N. Yamoah, Jennifer Kersigo, Karen Elliott, Niya LaRoda, Gabriela Pavlinkova, Bernd Fritzsch

**Affiliations:** ^1^ Department of Translational Neurosciences College of Medicine, University of Arizona Phoenix Arizona USA; ^2^ Department of Biology The University of Iowa Iowa City Iowa USA; ^3^ Department of Neurological Sciences University of Nebraska Medical Center Omaha Nebraska USA; ^4^ Laboratory of Molecular Pathogenetics, Institute of Biotechnology CAS BIOCEV, Center of Excellence Vestec Czechia

**Keywords:** cochlear neurons, cochlear nuclei, development, hair cells, vestibular neurons, vestibular nuclei

## Abstract

**Background:**

Knockin mouse models expressing calbindin (*Calb1*), calretinin (*Calb2*), and peripherin (*Prph*) exhibit changes in hair cells (HCs), spiral ganglion neurons (SGN), vestibular ganglion neurons (VGNs), and their central projections.

**Results:**

Developing cristae HCs show strong Calb1‐positive expression, but adult HCs are mainly Calb2‐positive. Utricle and saccule initially have Calb2‐positive HCs and later develop Calb1‐positive HCs in the striola region. Inner hair cells (IHCs) and outer hair cells (OHCs) in the cochlea express Calb2 early on. Calb1 expression in OHCs overlaps with Calb2; the expression of Myo7a, Calb1, and Calb2 reaches the apex later. SGNs and VGNs exhibit distinct Calb1 and Calb2 patterns but include a subpopulation with mixed expression. Central fibers are Calb1‐ and Calb2‐positive early in the developing cochlear nuclei (CN) and vestibular nuclei (VN) but remain highly Prph‐positive. VGNs innervate the lateral and VN, which are positive for Calb2 and Prph. Distinct Calb1‐positive neurons overlap with the anterior (A) and ventral (V) cochlear nuclei (AVCN, PVCN) with Calb2, while the dorsal cochlear nucleus (DCN) shows segregation of Calb2 and Calb1.

**Conclusion:**

We offer insights into the timing of how neuronal identity and connectivity are regulated in the auditory and vestibular systems, as shown by the expression of Calb1, Calb2, and Prph.

## Graphical abstract

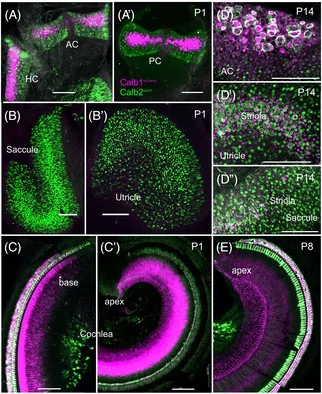

Three populations of hair cells have a distinct expression of Calb1 and Calb2. (A, A′D) The central is highly positive for Calb1 while surrounding HC are positive for Calb2. Later, a calyx forms primarily with Calb1. (B, B′, D′, D″) Saccule and utricle start out positive for Calb2 but will upregulate the Calb1 in the striola that is primarily forming calyx. (C, C′, E) Cochlear has a different progression of hair cells from base and apex. Note that at P8 there is an incomplete segregation from inner (Calb2) and outer hair cells (Calb1). Bar indicates 100 μm.

Key Findings

- hair cells, vestibular neurons, cochlear neurons, vestibular nuclei, cochlear nuclei, development.

## 1 INTRODUCTION

The complex perception of sound and head movements, along with responses to body position and gravity, depends on a diverse collection of cochlear and vestibular hair cells (HCs), spiral ganglion neurons (SGNs), and vestibular ganglion neurons (VGNs). The VGN and SGN axons project and form synapses at distinct and diverse nuclei in the brainstem, the vestibular nuclei (VN) and cochlear nuclei (CN), where encoded sensory information is further integrated.^1^, ^2^ An extensive sequence of gene expression is required to develop cochlear and vestibular HCs and their afferent neurons (SGN, VGN), which make synaptic connections at the VN and CN.^3^, ^4^ Distinct encoding proteins act as signature markers that can be used as tools for cell lineage tracing, serving as developmental and functional fingerprints. A large family of calcium (Ca^2+^) binding proteins features a helix–loop–helix signature EF‐hand motif.^5^ About 250 Ca^2+^ binding proteins can be separated into Ca^2+^ sensors. For instance, Ca^2+^ binding proteins and modulators, such as parvalbumin (Pvalb), calbindin (Calb1), calretinin (Calb2), and oncomodulin (Ocm), play a unique role as buffer proteins in the inner ear, exhibiting cell‐specific expression and distinct functions.^6^, ^7^ Calb1 and Calb2 expressions have been assigned to HCs and distinct cochlear neuron subtypes.^8^, ^9^ Genetic deletion of *Calb1*, *Calb2*, and *Pvalb* results in excessive exocytosis during prolonged depolarization in cochlear HCs.^10^, ^11^ Although it is unknown how vestibular HCs function without one or more of the three proteins.

Vestibular and cochlear HCs are diverse, as shown by studies using antibodies against Calb1, Calb2, and Pvalb in the canal cristae.^12^, ^13^, ^14^ Results show a differential distribution of Calb1 and Calb2 in type I and type II HCs.^15^ Moreover, a longitudinal study suggests a dynamic expression of Calb2,^16^ with incomplete overlaps of Calb1 and Calb2 within calyces in canal cristae.^13^ Multiple descriptions of utricular and saccular HCs show Calb1 and Calb2 expression, but information is scarce on Pvalb‐labeling.^12^, ^16^, ^17^, ^18^ A significant distinction between canal cristae and otoconia‐bearing end organs is the striola HCs, which are positive and overlap for Calb1 and Calb2. At the same time, Calb2 is positive for nearby extra‐striolar HCs.^13^, ^19^, ^20^ Despite a comparative insight into these studies,^15^, ^19^ the dynamic changes in cell‐marker expression are challenging to capture using antibodies.^16^, ^21^, ^22^ Like vestibular HCs, Calb2 shows a dynamic expression in the developing mouse cochlear HCs, showing sustained positivity for IHCs but transient positivity for outer hair cells (OHCs).^16^ In contrast, Calb1 developmental expression is unknown.^14^, ^23^

VGNs and SGNs are widely distributed among the five sensory organs and the cochlea. The VGN is divided into superior (sVGN) and inferior (iVGN) to innervate the vestibular HCs.^24^, ^25^ The cristae are innervated without fiber segregation from the cerebellum and the brainstem.^26^ In contrast, the utricle and saccule receive selective input from the cerebellum and the brainstem.^27^, ^28^ The sVGNs innervate the anterior (AC) and horizontal canal (HC) with the utricle and part of the saccule, and the iVGNs innervate the posterior canal (PC) and the remaining saccule. Moreover, three VGN subtypes terminate as calyces, dimorphic, and boutons afferents.^29^, ^30^ For the SGNs, there is a unique innervation of Calb1‐, Calb2‐, and Prph‐positive neurons that selectively innervate the IHCs and OHCs.^31^, ^32^ Moreover, in the cochlea, there is a place‐map longitudinal innervation forming a characteristic tonotopic design.^4^

VN and CN are innervated by fibers from the vestibular and cochlear nerves (VN, CN), which project centrally to different longitudinal and dorsoventral regions of the brain.^33^ The VN is derived from more rhombomeres between r1/cerebellum and the uvula and nodulus, extending beyond rhombomere 9. These rhombomeric columns r2–3 give rise to the superior VN, r4 to the lateral VN,^34^, ^35^ r5–r8 to the medial VN, and r5–r9 to the descending VN (or the spinal cord). The diverse origins of the auditory and vestibular neurons suggest multiple subtype markers are likely, as demonstrated for SGNs.^31^, ^36^, ^37^ However, potential dynamic changes are challenging to capture with antibody labeling. In contrast to nine rhombomeres of the VN, three CNs are formed between rhombomere 2 and 5: the anteroventral, posteroventral, and dorsal CN (r2–3, AVCN; r4, PVCN; r5, DCN). The origin of the CN depends on two gene expressions: *Atoh1* and *Ptf1a*.^4^, ^35^, ^38^, ^39^, ^40^, ^41^ The structural topology of the cochlea is preserved such that the basal and apical SGNs project to the dorsal and ventral aspects of the CN, respectively.^4^ Using three antibodies against Calb1, Calb2, and Pvalb, Lohmann and Friauf^42^ described the central projection but showed an incomplete input from cochlear fibers. The topology of fibers connected to specific CN remains unclear at the intersection.

We used mouse models expressing Calb1‐mCherry, Calb2‐eGFP, and Prph‐eGFP to determine the dynamics of neuronal identity in auditory and vestibular HCs, sensory neurons, and their central projections. Our findings not only confirm earlier reports but also reveal novel interactions between cochlear and vestibular HCs, the spiral and vestibular ganglia, and the cochlear and VN.

## 2 RESULTS

### 2.1 Hair cells development

Vestibular HCs are the first to differentiate, marked by *Prox1* upregulation in the canal cristae about E11.5.^43^ Their differentiation continues in an inside‐out pattern of *Atoh1* upregulation starting around E13.5.^44^, ^45^ The generation of new vestibular HCs continues beyond birth at least until P14.^46^, ^47^ In contrast, cochlear HC differentiation begins around E12.5, coincident with *Prox1* expression, followed by *Atoh1* upregulation at about E14.5,^43^, ^45^ and in the cochlear middle turns, progressing bidirectionally toward the base and the apex, reaching the apical tip much later, around P2.^48^ Thus, while vestibular HC differentiation follows an inside‐out pattern, cochlear HC differentiation follows a complex longitudinal expression toward both the base and apex.

A significant difference between vestibular and cochlear HCs lies in their innervation pattern and functional organization. Vestibular HCs are classified into three types based on their innervation patterns: calyx, bouton, and dimorphic.^29^ In contrast, cochlear HCs differentiate into two types: a single row of IHCs and three rows of OHCs.^49^ Moreover, the auditory system is tonotopically organized, encoding sound from high to low frequencies along the cochlear base–apex axis, with topographically restricted SGNs to discrete iso‐frequency bands.^4^, ^50^ No such topological organization exists in the vestibular HCs. Instead, the utricle and saccule show a counter‐organization, providing an opposing polarity within the maculae,^26^, ^28^ while all HCs in the cristae have a uniform directional organization. With this background, we clarified the potential relationship between vestibular and cochlear HCs across development.

### 2.2 Vestibular and cochlear hair cells: their development and formation

#### 2.2.1 Canal cristae hair cells

**FIGURE 1:**
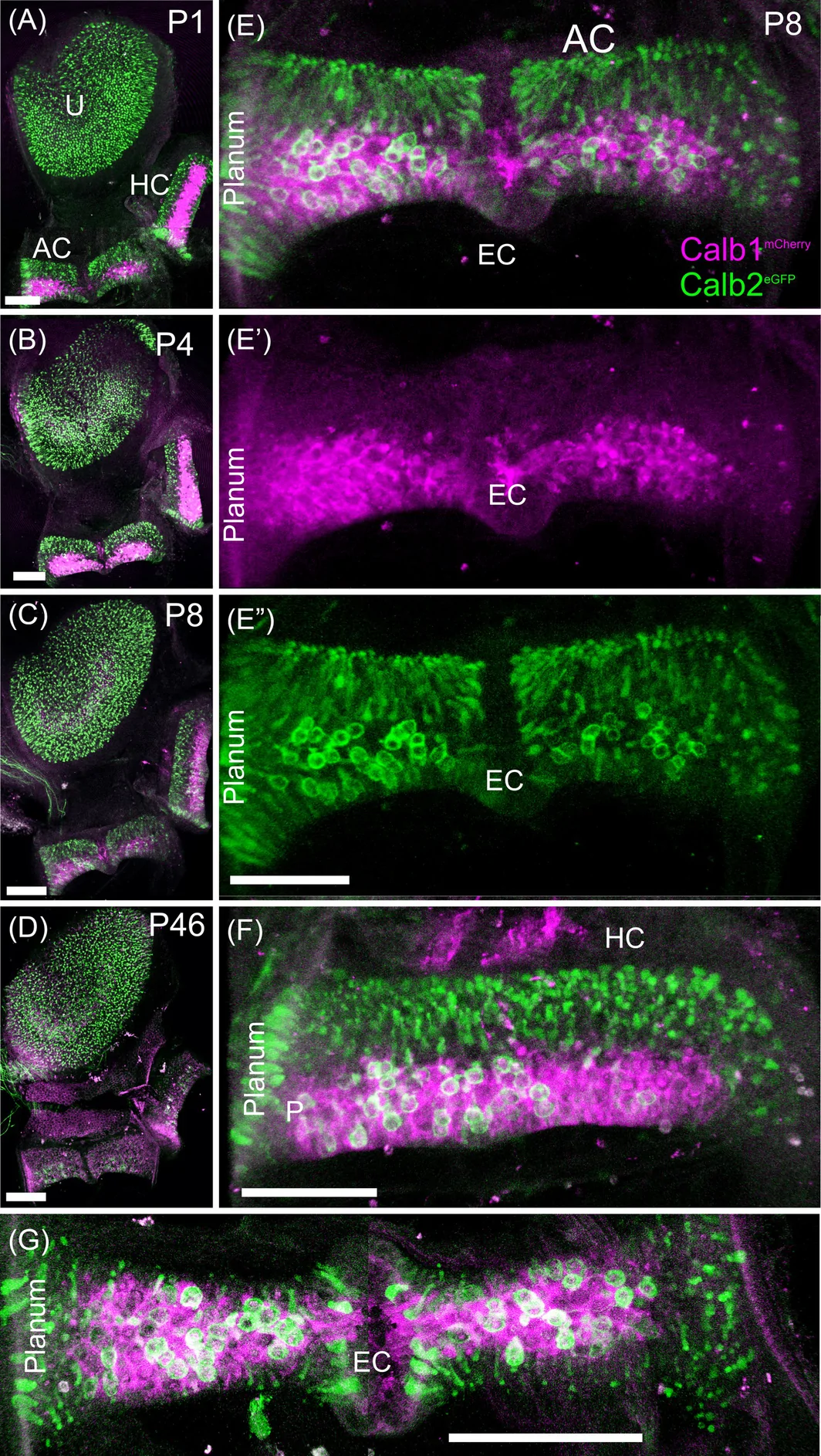
The development of the canal cristae shows a meaningful change in Calb1 and Calb2 expressions. The strongest expression is Calb1, which labels the cristae, but the expression declines with age (A–D). In contrast, the utricle and saccule start as highly positive for Calb2, which later adds Calb1 in the striola. The distribution in the eminentia cruciatum (EC; absent in the HC; F) shows strong positivity for both Calb1 and Calb2, which do not overlap. The planum is formed bilaterally (except for the HC, E, F, and G) and starts highly positive for Calb2, which will be replaced by Calb1 (compare C, D). Calb2 positive forms as calyx at about P4–8 (E–G), showing double and straightforward calyx with an occasional triple calyx. Boutons are below the resolution for light microscopy. The bar shows 100 μm.

HCs in the three canals provide the proper three‐dimensional body orientation information: the AC, HC, and PC. A highly elevated level of Calb1 is seen in the earliest stages of development (P1–P8; Figure 1A–C), but this level gradually reduces by P28 and P46 (Figures 1D, S1 and S3, Supporting Information). Moreover, an asymmetric Calb1 expression is seen at the abneural crista in AC compared with the neural cristae aspects (Figures 1B and S1). The eminentia cruciatum at the AC and PC cristae is weakly positive for Calb1 for both HCs and supporting cells (Figures 1E, E′ and S1). From P1–P8, the planum (Figure 1) shows a progressive upregulation for Calb1‐positive cells, as seen in older mice (P28, P46). The expression of Calb1 has the exact orientation of the planum in anterior and horizontal cristae (Figure 1E, E′, F) but has a different orientation parallel to the PC (Figure 1G). Note that the planum forms only on one side in the developing horizontal crista (Figure 1E, F). The Calb1 expression is higher in the AC and HC abneural aspects than in the nearby PC (Figure 1E–G). Moreover, one planum is further upregulated in the canal cristae at P28 and P46 (Figure 1D), while the Calb1 expression is reduced to the background in the canal cristae. In summary, there is an asymmetric expression of Calb1 in the cristae, favoring the planum on one side in developing and older mice.

In contrast to Calb1, Calb2 expression onset in HCs^22^ is at the planum (P1–P8; Figure 1A–C). In addition, the calyx formation begins near the planum at and between P4 and P8 (Figure 1C, E, E″, F), and there is gradual maturation in older mice (P28–P46). This upregulation of calyx formation is notably different in the horizontal cristae at P8 (Figure 1F). Moreover, it overlaps with Calb1 expression in the abneural part of the canal cristae. At the same time, the neural section is free of calyx formation (Figure 1E, E″, F). Quantification of calyx formation shows ~17–23 (±3.5, SD) cells in the abneural planum, while only 3–10 (±2.3, SD) non‐planum cells are found in P4–P8 old mice. In addition, the innervation pattern was biased, showing more Calb1 HCs next to the planum, while the non‐planum section had fewer or hardly any Calb1 HCs. In P28 and older mice, the distinction of the planum was equal and had more calyx‐bearing HCs (Figure 1). From the level of Calb1, many HCs showed a bias toward the planum. Compared to Calb2, it clearly shows the overlap of the calyx, which also reveals Calb1‐positive HCs in the calyx, as seen in P8. Consistent with the early calyx formation, it correlates with the larger fibers compared to the nearby utricle (Figure S1). Fibers that innervate the canal cristae showed Prph‐positivity, which are thin fibers compared to inner ear efferent (IEE) efferent (Figures 1 and S1).^9^, ^51^, ^52^

#### 2.2.2 Utricular and saccular hair cells

**FIGURE 2:**
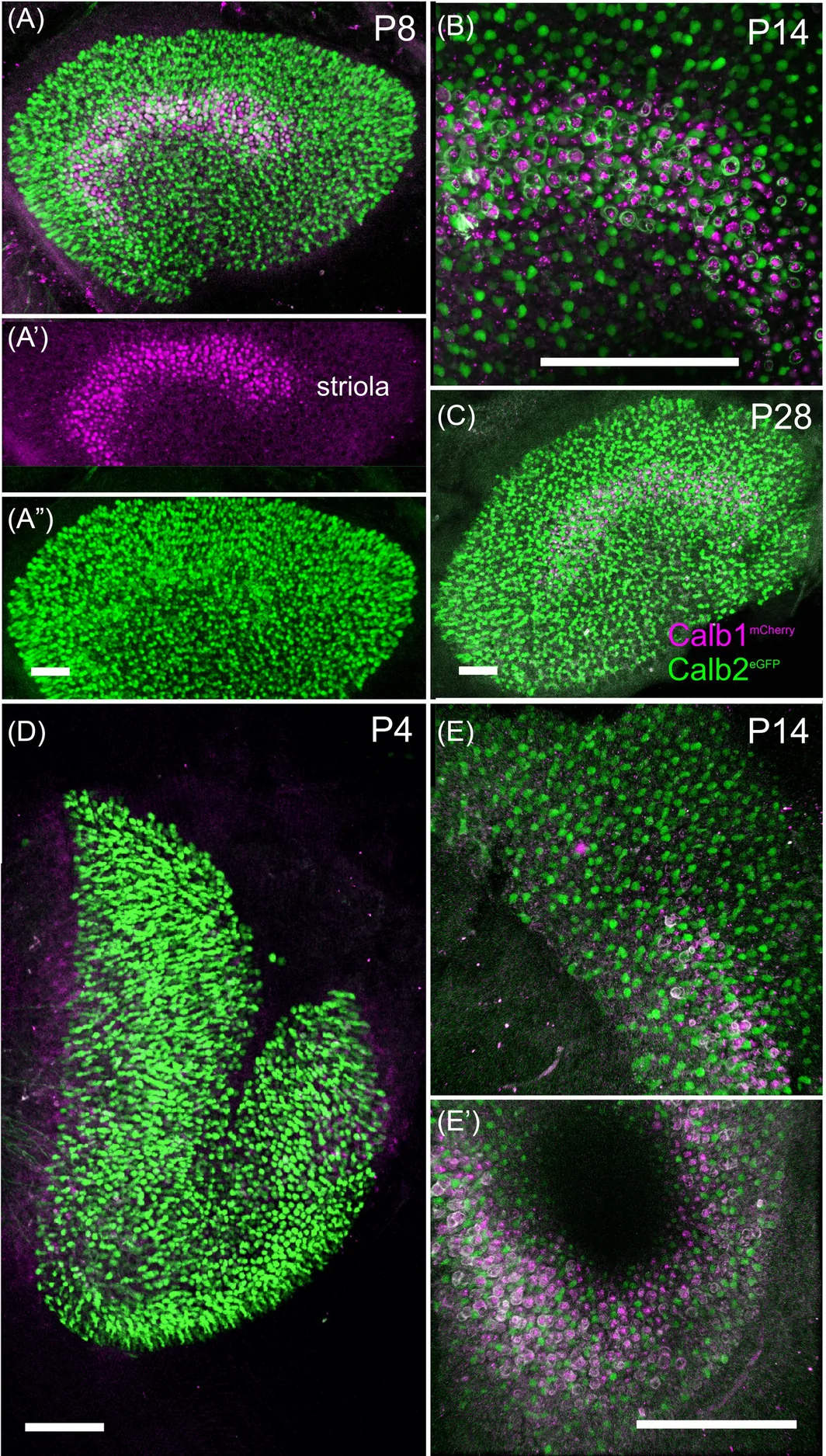
Utricle and saccule show a distinct Calb1 and Calb2 expression. The strong Calb2 is about P4 in the utricle and saccule (D). At P8, the striola appears to be positive for Calb1 (A). This makes a distinction of a boundary (A–A″) that stays positive in the striola in older mice (B, C). A unique positivity is a distinct population between Calb1 and Calb2 that mostly segregates from each other (B). Note that Calb1‐positive hair cells are mostly forming in the calyx (B, E, E′). The bar shows 100 μm.

Calb1 is absent in both the otoconia‐bearing end organs, the utricle and the saccule, before birth, but begins to be expressed at ~P4, persisting in adults (Figure 1). There is limited Calb1 expression at the striola as documented for P8 and older mice (Figure 2A, C, D). In contrast, Calb2 is highly positive in nearly all HCs that are interlaced with Calb1 in the striola (Figure 2B insert). A significant difference is observed in calyx formation. Calyx formation first occurs in the canal cristae at about P8 (Figure 1C, E, F), as reported using antibodies.^22^ However, calyx formation in HCs of the utricle and saccule occurs between P8, and P14 (Figure 2B, E, E′), and P28, which is slightly later than what was reported using antibody detection.

We verified the Calb1/Calb2 expression using anti‐Calb1 and anti‐Calb2 antibodies (data not shown). Calb2 is expressed in all vestibular HCs, except in the striola region, where Calb1 is upregulated later. Within the positive HCs, single and double calyx‐type nerve endings are identified that are mildly positive for Calb1 in the canal cristae (Figures 1, 2 and S1) and show distinct labeling for either Calb1 or Calb2 in the striola (Figure 2A, A′, B, B′). Notable is the late upregulation for Calb1 that forms primarily the calyx formation in the striola. The boundary for Calb1 allows us to distinguish the striola region between the Calb1 and Calb2‐positive HCs to provide details of striola and extra striola expression. Prph‐positive fibers innervate the canal cristae, utricle, and saccule, but do not reach the striola (Figure S1).^30^, ^51^

#### 2.2.3 Cochlear hair cells

**FIGURE 3:**
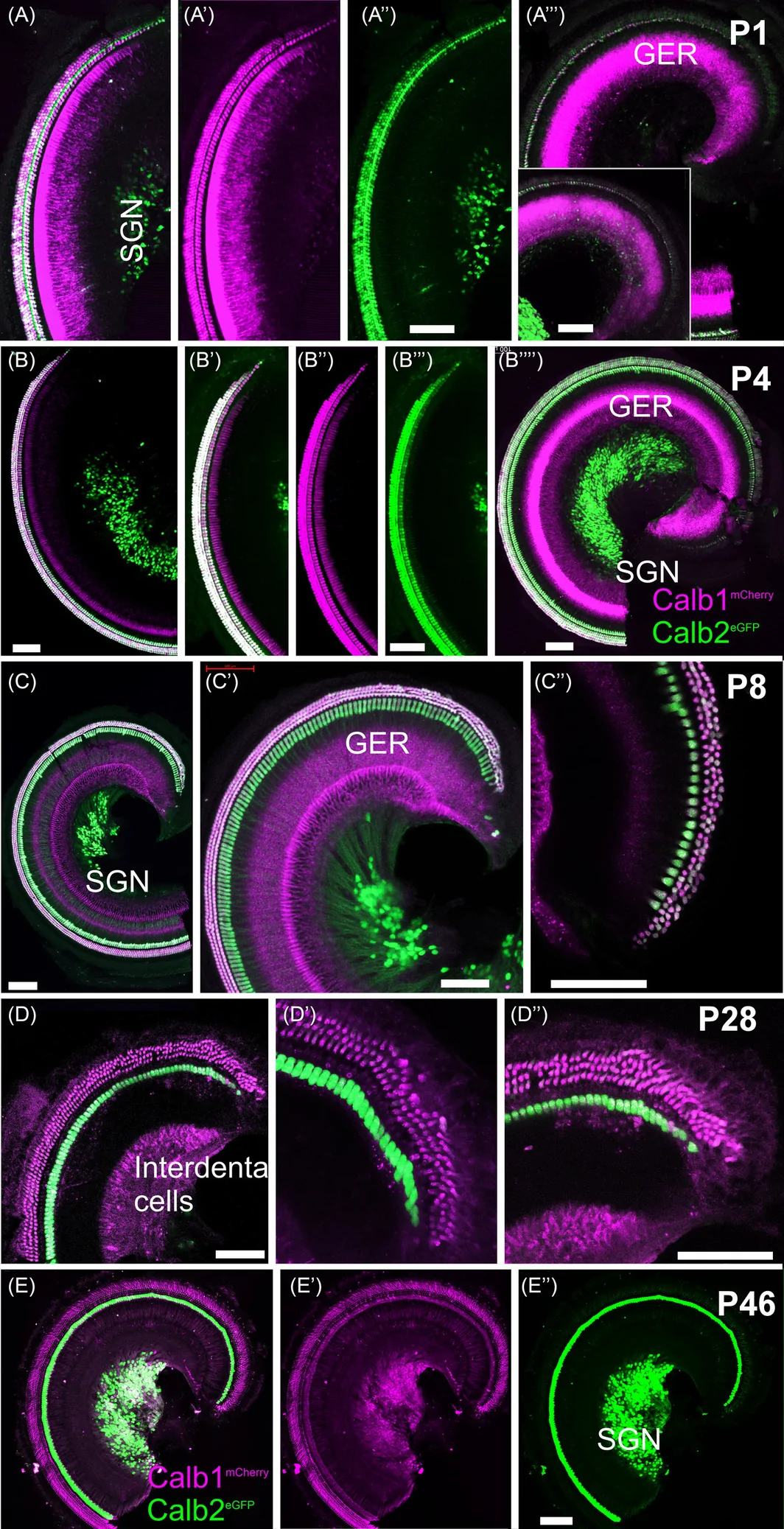
OHC and IHC expressions. Upregulation of Calb1 and Calb2 shows an incomplete overlap for the base and the apex (A–A⁗). Note that a gradient is positive in the base and the apex, which begins in the IHC, is positive for Calb2. By P4, both Calb1 and Calb2 are in OHC and IHC at the base (B–B‴). At the apex, Calb2 is leading but will be incompletely positive for the upper middle turn for Calb1/Calb2 (B⁗). Note the progression of Calb1, which is initially positive for the GER but eventually becomes lost. The expression in interdental cells shows an upregulation of Calb1 (A‴, B⁗, C′, D). At P8, Calb1 and Calb2 in OHC and IHC (C–C″). The cochlear base is a much longer IHC that will eventually end at OHC (B–B‴), while the OHC extends beyond the IHC in the apex (C″), which will eventually add a single IHC. In addition, the OHC varies in the apex between 2 and 4 (D–D″). It takes about P46 to reach out to the adult distribution, which is primarily positive for OHC (Calb1), exclusively positive for IHC for Calb2, and has limited positivity for IHC for Calb1. The bar shows 100 μm.

Calb1 is strongly expressed in the greater epithelial ridge (GER) between E18 and P4 (Figure 3A, B). The GER expands toward the base (Figure 3A, B) while showing a delay at the apex (Figure 3A, B′). As the GER disappears between P8 and P14, its remnants can be seen, becoming the inner spiral sulcus (Figure 3C). The Calb1 longitudinal expression starts in the middle turn in IHCs and OHCs (Figure 3) but follows different developmental timelines. Calb1 expression reaches the end of the cochlear basal turn by ~P4 (Figure 3A, B) and the apical turn by ~P8. In the adult cochlea, Calb1 expression is significantly reduced at the basal turn IHCs, with only faint expression remaining in apical IHCs (Figure 3E). Additionally, highly Calb1‐positive HCs are seen between IHCs, extending toward the ductus reuniens at P4. In contrast, Calb2 expression is nearly equal and overlaps with Calb1 in the OHCs (Figure 3A, B). Notably, the apex shows multiple rows of OHCs that are disorganized in expression pattern (Figures 3C′, D′, D–E″ and S2).

**FIGURE 4:**
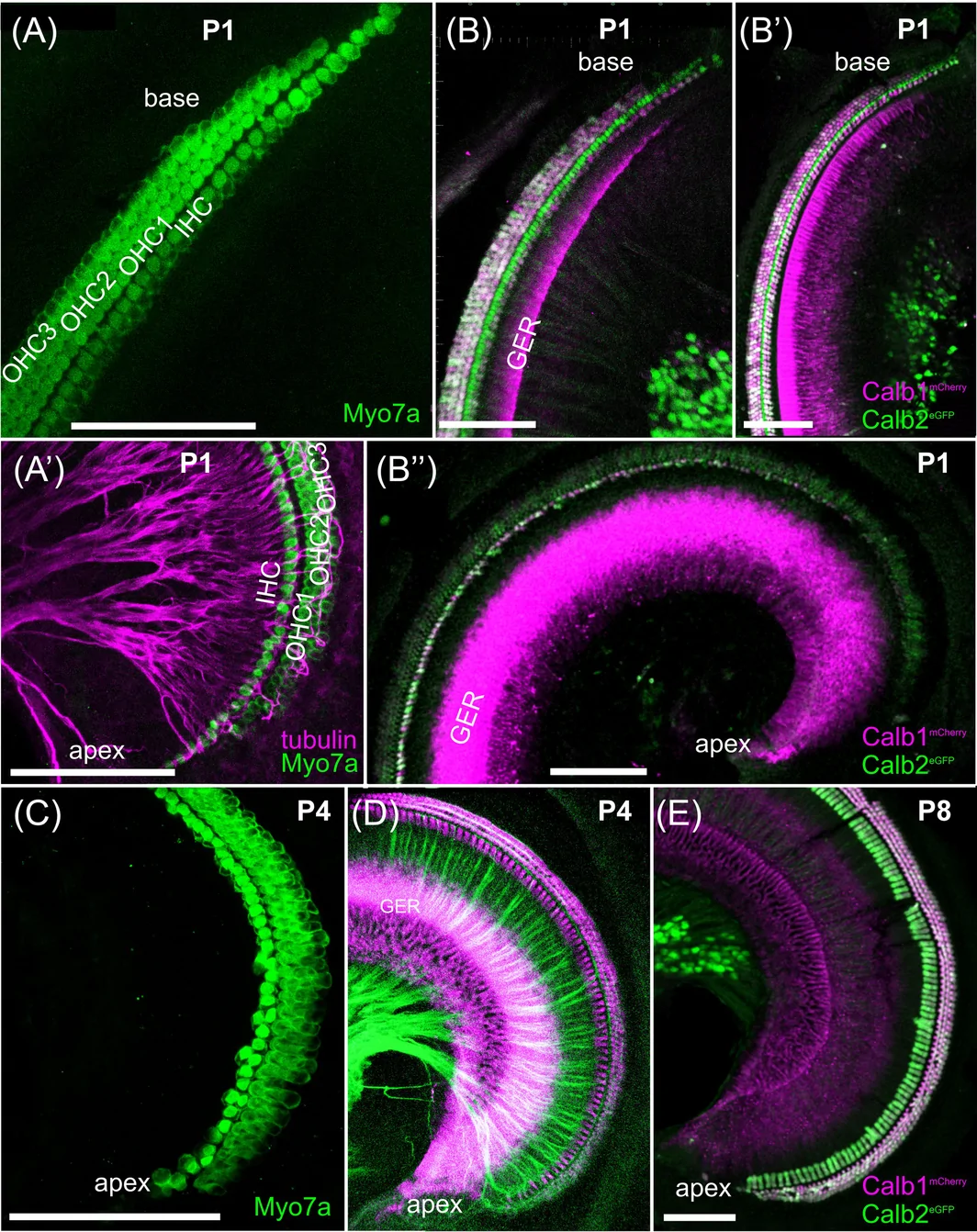
Upregulation is earlier positive for Myo7a compared to Calb1 and Calb2. Expression is positive for P1 in the base for Myo7a, Calb1, and Calb2 (A, B, B′), while there is a delay for Myo7a (A), but there is a significant delay in Calb1 and Calb2 expression in the apex (B″). At P4, nearly all apical HCs are positive for Myo7a (C), which still has a delayed expression of Calb1 and Calb2 (D). Even at P8, old mice show an overlap of Calb1 and Calb2 in the apical tip that is already either positive for the IHC (Calb2) or OHC (Calb1), more medial (E). The bar shows 100 μm.

Closer examination of Myo7a expression shows strong positivity in all HCs in the base at P1 (Figure 4A). While the first row of OHC is positive at the basal tip, gaps are present in the middle row, while the tip of the base is free from Calb1, showing a clear difference with Calb2 (Figures 3A and 4B, B′). In contrast, the apex of P1 shows a delayed upregulation for either Myo7a (Figure 4A′) and an even more pronounced delay in Calb1 or Calb2 expression (Figures 3A′ and 4B″). By P4, the apical tip is positive for Myo7a (Figure 4C), yet it still lacks Calb1 or Calb2 expression (Figure 4D). The apical tip follows the same OHC progression as the base (Figure 3A, B). Even at P8, OHCs have a mixed distribution of Calb1 and Calb2 (Figure 4E). However, by P28 and in older mice, Calb1 levels in IHCs are slightly reduced, while Calb2 is barely detectable in OHCs (Figures 3D, E and S2). In summary, the longitudinal progression of marker expression begins in the middle cochlea, reaching the base by P1, while the apex shows a delayed expression of Myo7a, Calb1, and Calb2.

#### 2.2.4 Connecting afferents with hair cells

**FIGURE 5:**
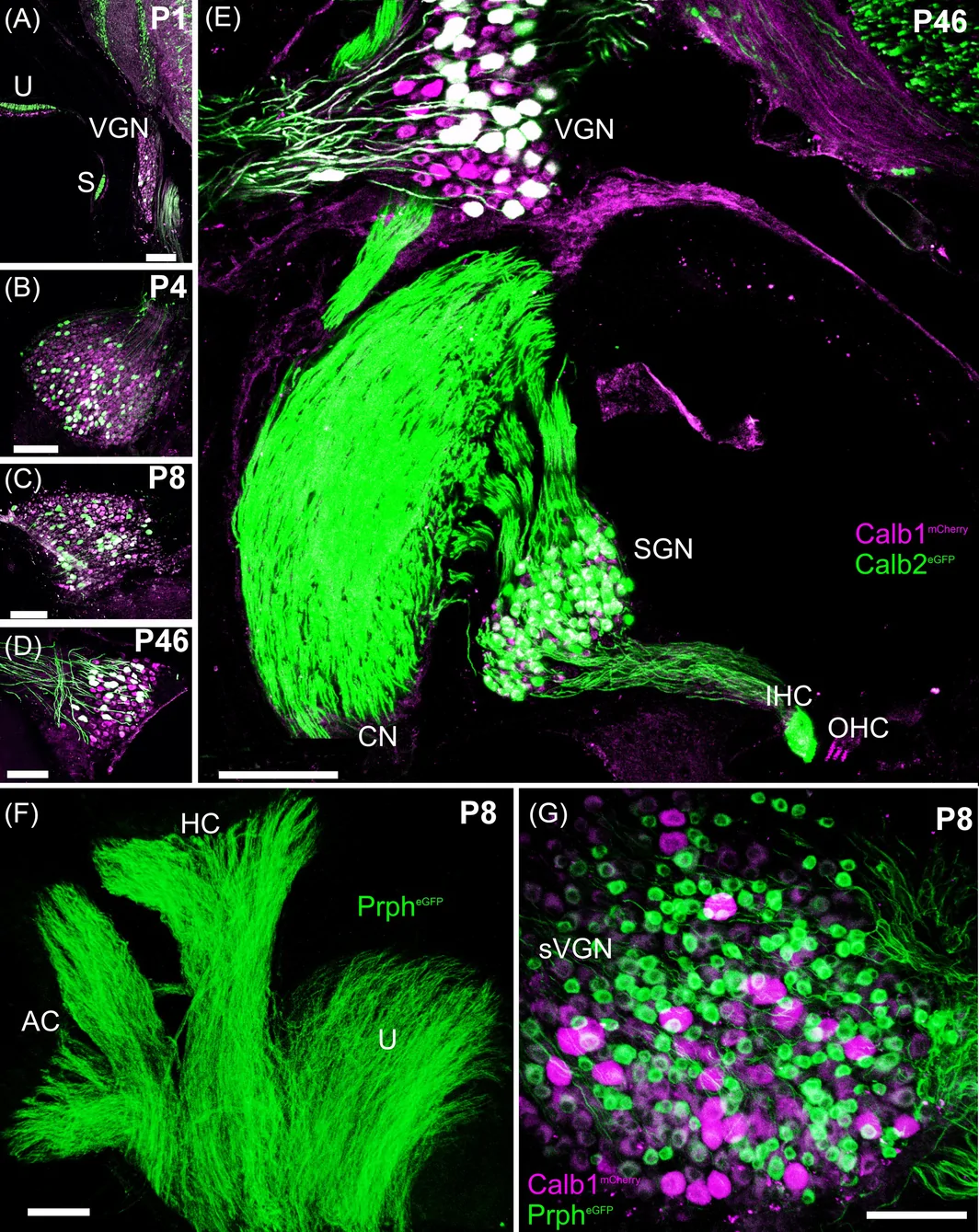
Vestibular and cochlear neurons are positive for Calb1, Calb2, and Prph. In this section, we show the progression of VGN that starts in the Calb1 (A) and eventually adds more Calb2‐positive neurons (B–E). The largest neurons are positive for Calb2, which is nearly as large as Calb1 (E). Compared to VGN, SGN is more positive for a much smaller Calb2 that overlaps with the less favorable Calb1. Note the distinct IHC that are highly positive for IHC, while OHC is positive for Calb1. A whole mount shows the smaller Prph neurons (G), followed by slightly larger but faintly Prph‐positive. Fiber from the VGN can be seen exiting from the sVGN to innervate the canal cristae, utricle, and saccule (F). The bar shows 100 μm.

Prph‐positive fibers project to innervate OHCs (Figure S2).^32^, ^53^ Calb1 shows mild positivity in nerve fibers reaching out to both vestibular and cochlear HCs (Figure 5E). In contrast, Calb2 is highly expressed in fibers innervating the calyx of the canal cristae, saccule, and utricle but is restricted to older IHCs in the cochlea (Figures 1E–G and 4A, E). Prph‐positive fibers innervate the utricle, saccule, and canal cristae (Figures 5F and S1). Detailed innervation patterns show a simple connection of boutons on vestibular HCs, with Prph‐positive branches extending along the perimeter but also innervating the middle part of the saccule, utricle, and canal cristae.

Calb2‐positive fibers have vastly different distributions for vestibular and spiral ganglion processes. A small subset of fibers innervates the canal cristae, forming Calb2‐positive calyces (Figure 1E–G). Slightly thinner fibers innervate the utricle and saccule but do not reach the striola. However, the innervation develops between P8 and P28, and no calyx structures can be shown in the saccule and utricle within the striola at P14. Consistent with previous studies in adult animals,^15^, ^19^ this confirms the formation of single, double, and triple calyces.

## 3 VESTIBULAR AND SPIRAL GANGLIA DEVELOPMENT

The vestibular ganglia begin to develop around E9,^3^, ^54^ while the spiral ganglion develops later, between E10 and 12.5.^31^, ^55^ The vestibular ganglia split into two populations (inferior, iVGNs and superior, sVGNs), while the spiral ganglion progresses from the base to the apex of the cochlea.^56^ Consequently, VGNs are incompletely segregated among the five end organs, while a longitudinal segregation of SGNs in the cochlea provides the tonotopic organization.^4^ We used tracing with lipophilic dyes to show the distribution of vestibular neurons that overlap with Calb1, Calb2, and Prph.

### 3.1 Vestibular neurons form distinct populations that segregate into the superior and inferior VGN

**FIGURE 6:**
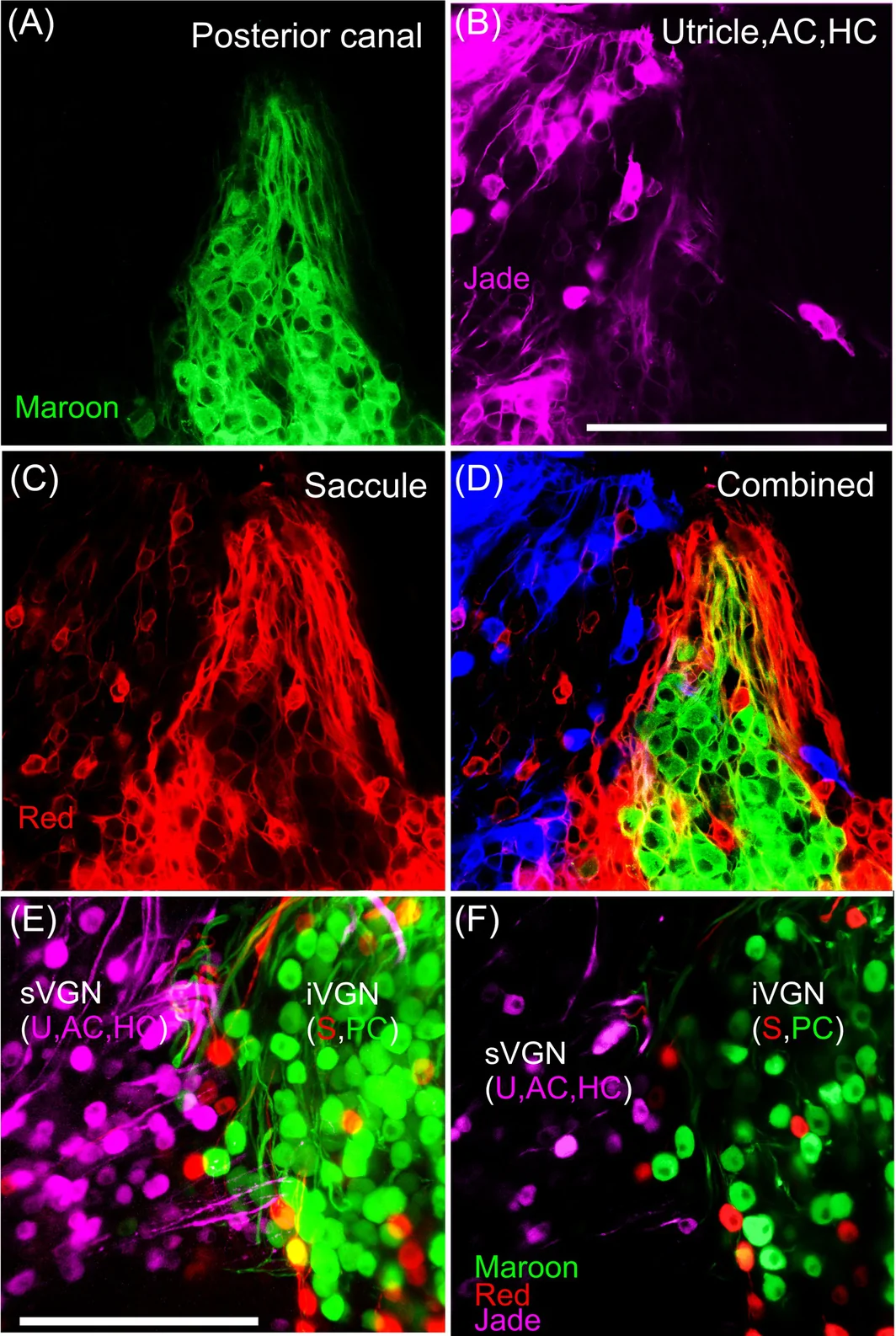
Tracing dyes reveal incomplete overlap from VGNs. A triple labeling method, which labels the PC (green, A), the utricle (anterior, AC), and horizontal canal (HC) (jade, B), and the saccule (red, C), can document the distinct and incomplete overlap (D) in P2 old mice. The whole mount (E) and optical section (F) show a unique segregation from the posterior canal (PC, maroon) from the utricle, AC, and HC (jade) that splits between the saccule (red, C, F). The bar shows 100 μm.

Our analysis shows incomplete segregation of the superior and inferior vestibular ganglion (sVGN and iVGN, Figures 6 and S3). Triple‐color dye tracing from the utricle, ACs, and HCs shows projections to the sVGN, while a distinct labeling pattern from the saccule and PC marks a separate population from the iVGN (Figures 6 and S4). Additionally, a distinct population of neurons is positioned at the border between sVGNs and iVGNs, with the projections from the saccule (Figure 6). The boundary between the sVGN and iVGN is well defined for the utricle, AC, HC, and PC, but remains less clear for the saccule, except for a minor exception (Figures 6 and S4). The majority of VGNs innervate the AC, HC, and PC, while a smaller population projects to the utricle and saccule.

#### 3.1.1 Calbindin expression pattern in VGNs

**FIGURE 7:**
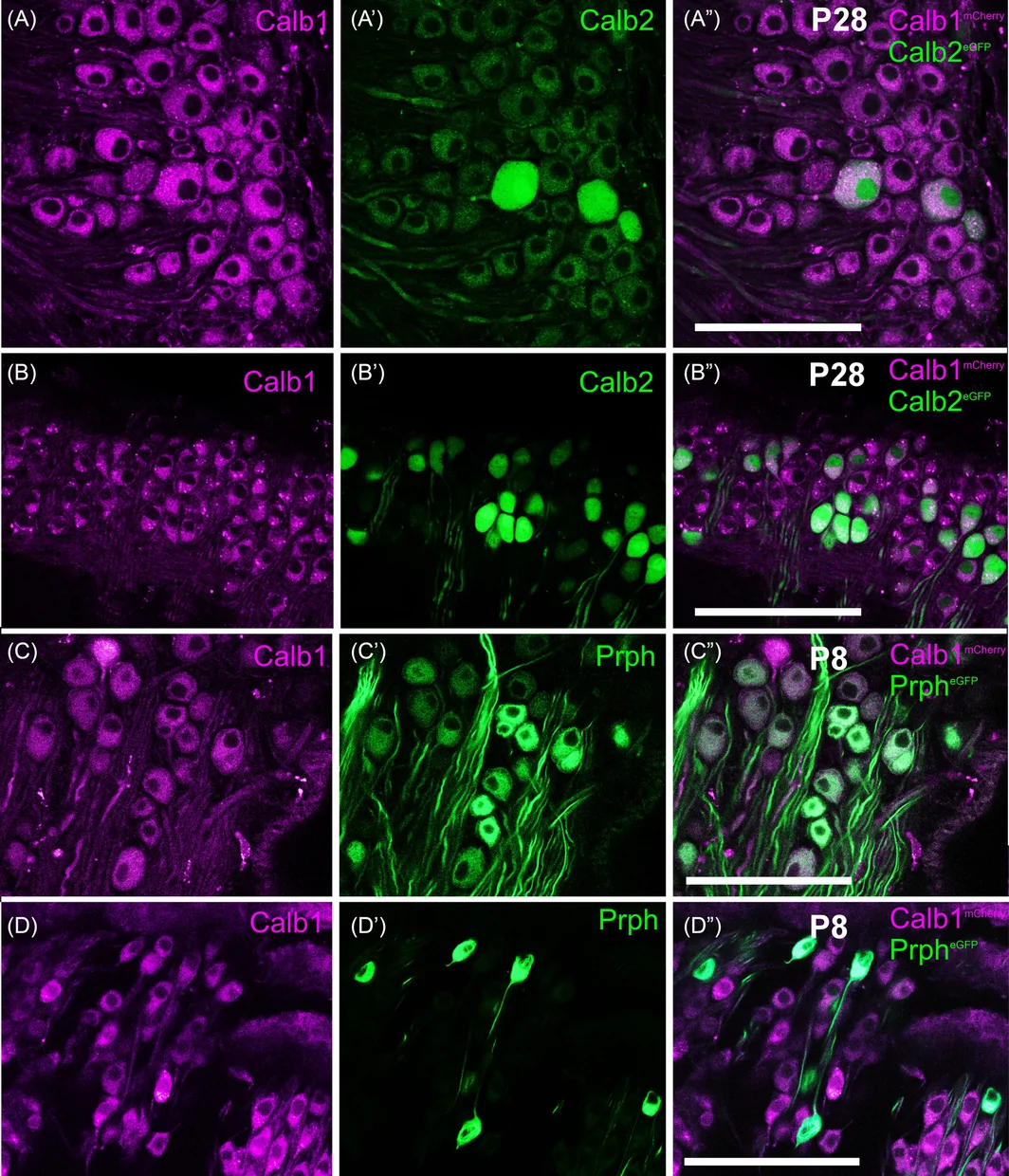
Calb1, Calb2, and Prph have distinct and largely overlapping functions in spiral ganglion neurons. Using whole mounts of P28 (A, B) and P8 (C, D) shows a different expression level. Nearly all neurons are positive for Calb1, with varying intensities ranging from strong to barely positive (A–D). In contrast, fewer neurons are positive for Calb2, which is highly positive for larger and medium‐sized neurons (A′, B′). Even the overall smaller neurons of Calb2 tend to be larger than Calb1, which is highly positive for the nucleus (A′, A″, B′, B″). In contrast, among the larger neurons that are positive for Calb2, the smallest neurons are the most positive for VGNs. However, the transient positivity is reduced to labeling exclusively the type II neurons that innervate the OHCs (C′, D′). Nearly all VGNs exhibit a variable intensity compared to Calb1, whereas certain SGNs, which are absent from Calb1, are highly positive for Prph (C″, D″). Bar shows 100 μm.

Initially, most VGNs are Calb1‐positive (Figure 5). However, we see a vastly distinct size variation of VGNs (Figure 7; Table 1). The smallest VGNs, measuring approximately 50–100 μm^2^, are the most frequent, while the largest can extend to 550 μm^2^. Cell size measurements were helped by the unstained nucleus (Figure 7; Table 1). Calb1 expression varies across VGNs, with little to no positivity in middle and large neurons. Only the largest Calb1‐positive neurons show weak fiber labeling without a clear resolution of the terminal branches (Figures 5 and S3; see also Figure S4).

**TABLE 1:** Quantitative neurons to VCN and SGN.

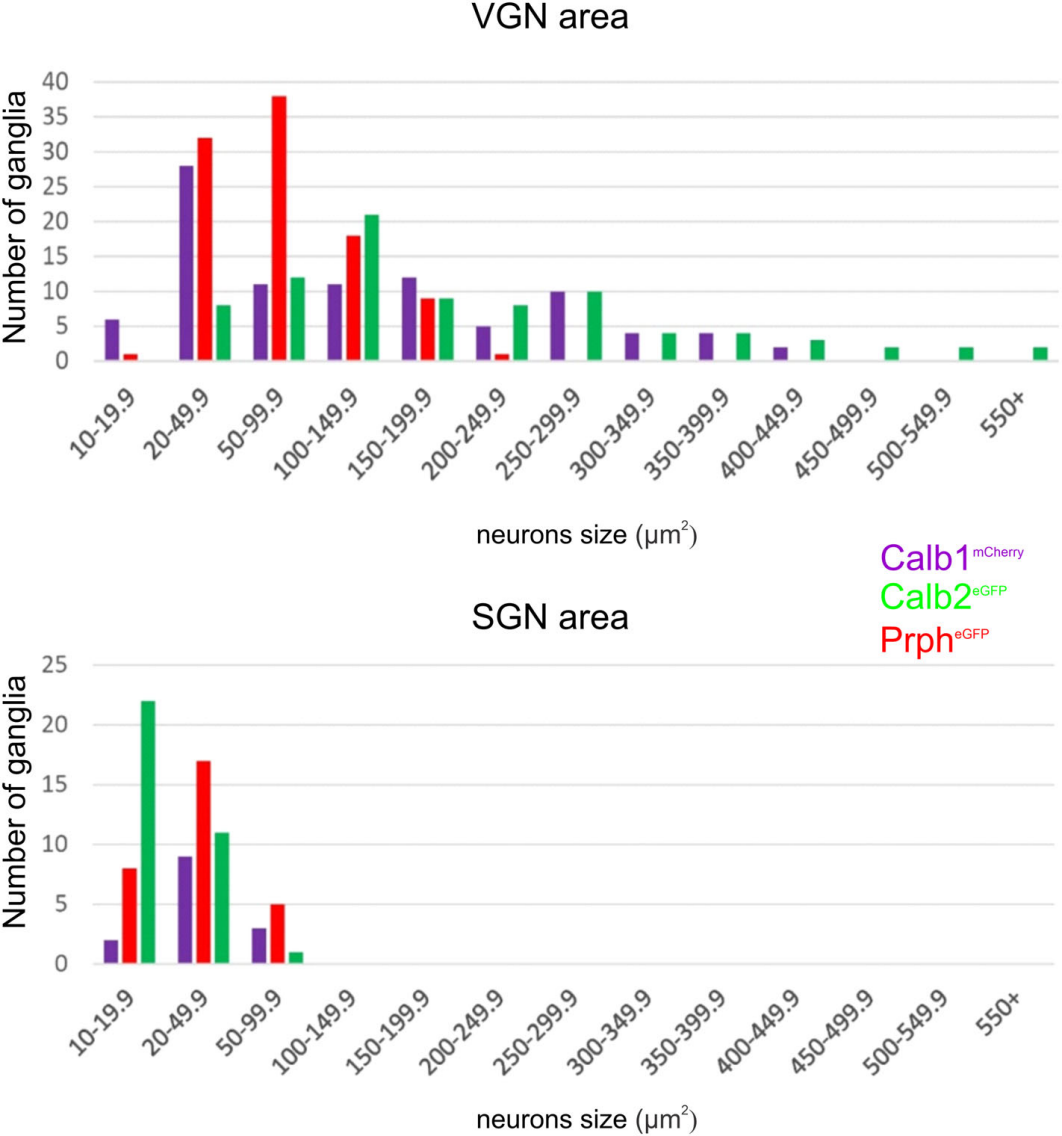

#### 3.1.2 Calretinin expression pattern in VGNs

A small population of VGNs is positive for Calb2; neurons primarily consisting of giant neurons, by size measurements, represent the largest neurons within the VGNs (Figures 5, 7 and S3; Table 1). In addition to the extremely high expression of Calb2, these neurons show high positivity in the nucleus compared to Calb1‐positive neurons (Table 1). Medium‐sized neurons are less likely to be Calb2‐positive than Calb1‐positive neurons.

#### 3.1.3 Peripherin is highly positive in the smaller VGNs

A large population of neurons is Prph‐positive, with fibers extending to the canal cristae, utricle, and saccule (Figures 5 and 7, S1 and S3). The Prph‐positive neurons are smaller on average and show extreme positivity around the unlabeled nuclei. Medium‐sized neurons show less intense Prph labeling, although it is present during early development. Nearly all VGNs are positive for Prph at birth (Figure 7, Table 1), but lower Prph expression in some neurons may account for the reduced number of VGNs shown in other studies.^12^, ^51^

### 3.2 Spiral ganglion neurons have a unique distribution compared to VGNs

Initially, nearly all SGNs are positive for Calb1, with very few exceptions (Figure 7 and S2). Compared to VGNs, SGNs are overall smaller in size (Figure 5; Table 1). Neuronal density varies depending on the cochlear location; the basal and middle turns have about the same density, while it tapers at the apex.^57^, ^58^ The whole mount shows that bundles of Calb2‐positive SGNs tend to be slightly larger than Calb1‐positive neurons (Figure 7). By P28, there is a clear distinction between Calb1‐ and Calb2‐negative SGNs. We have shown that during development, SGNs show Prph‐positivity.^32^, ^53^, ^59^ In older mice, double‐labeling for Prph and Calb1 reveals a distinct distribution, with a small population of Prph‐positive neurons that do not overlap with Calb1‐positive neurons. In addition, Prph‐positive neurons are among the smallest SGNs (Figures 7 and S2 and S3; Table 1).

#### 3.2.1 Double labeling for Calb1/Calb2 and Calb1/Prph

A distinct distribution pattern is evident in Prph‐positive neurons. In VGNs, Prph expression is highest in the smallest neurons, moderate in medium‐sized neurons, and limited in the largest VGNs. In contrast, during early cochlear development, most SGNs express Prph, but it becomes restricted to a small subset of neurons in the adult (Figure 7, S2, S3, S5, and S6; Table 1).

#### 3.2.2 The apical SGNs form a unique connection with the vestibulo‐cochlear neurons

**FIGURE 8:**
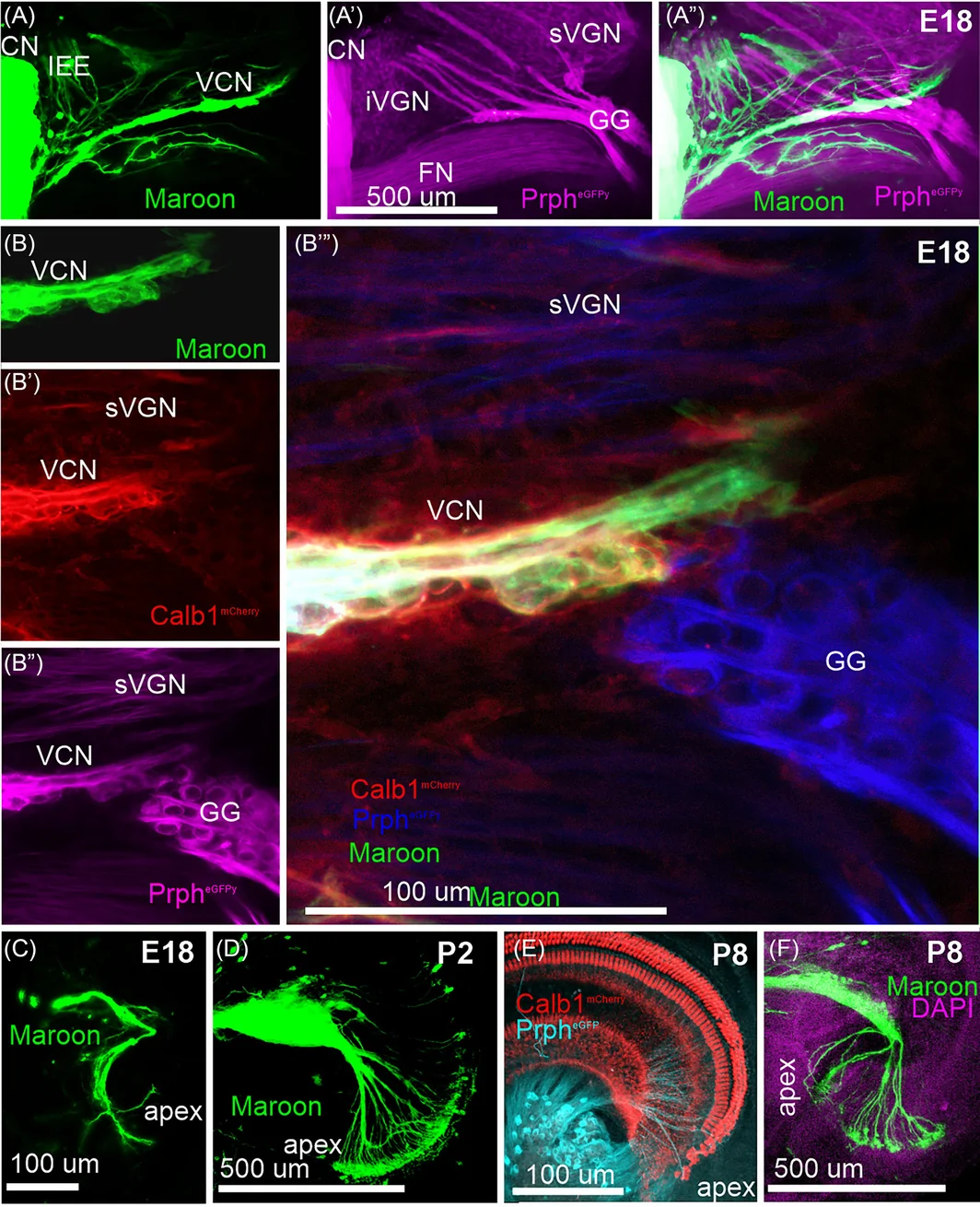
Apex connects to the vestibular‐cochlear neurons (VCN). Applied dyes trace (maroon, green) of the fibers to the CN (A), which also has a branch that runs parallel to VGN next to facial fibers (FN). A few fibers continue below the geniculate ganglion (GG, A′, A″). Using Prph (A′, B″, B‴) and Calb1 (B′) show that are positive in the VCN labeled that also labeled with dye tracing (B′, B‴). Inserting dye into the sVGN (see A″, B‴) labels selectively SGN neurons that innervate the tip of the apex (C, D, F) with maroon tracing (in green). The apex is highly positive for Prph, which innervates the tip of the apex, as shown by Calb1 (E). Note that Calb1 is labeled as red while Prph is either lilac, blue or cyan. The bar shows 100 and 500 μm.

Dye insertion at the apical tip reveals fibers that project to the CN, along with a unique population of branching fibers near the saccular fibers (Figure 8A).^60^, ^61^ This bundle is next to the VGN and converges later with the sVGN. In addition, inner ear efferent (IEE) and a small population near iVGNs are labeled (Figure 8A, A″). A dye insertion near the facial nerve also labels some neurons overlapping with the sVGNs, while the SGN population is selectively labeled from the apex (Figure 8C, D, F). What makes this unique is that it allows labeling of the bundle using Prph‐eGFP tissue (Figure 8A′, A″), which nearly converges at the geniculate ganglion (GG). Double‐labeled (lilac, green) after dye insertion at the tip of the apex reveals the detailed segregation of the GG from the VCN (Figure 8B‴ and S4). In addition, the same population is more prominent compared to nearby Calb1‐expressing neurons (Figure 8B′). A much larger population of neurons near the apex is labeled with dye insertion into the VCN, where most neurons are small, with a few larger neurons interspersed among them (Figure 8C, D, F). A highly Prph‐positive neuron shows a unique population that selectively extends toward the apical tip. At the same time, Calb1 is expressed in cochlear HCs (Figure 8E). Note that Calb1 was labeled as red while Prph was labeled as either lilac, blue or cyan while tracing is always green. Double labeling with distinct tracers also shows saccular neurons labeled from both iVGN and sVGN, projecting to the fibers and neurons of the VCN (Figure S4). In summary, using the three genetically modified mice and lipophilic dyes, we show a distinct and incompletely overlapping neuronal population with VGNs.

### 3.3 The central projection of vestibular and cochlear fibers results in different longitudinal and dorsoventral projections

**FIGURE 9:**
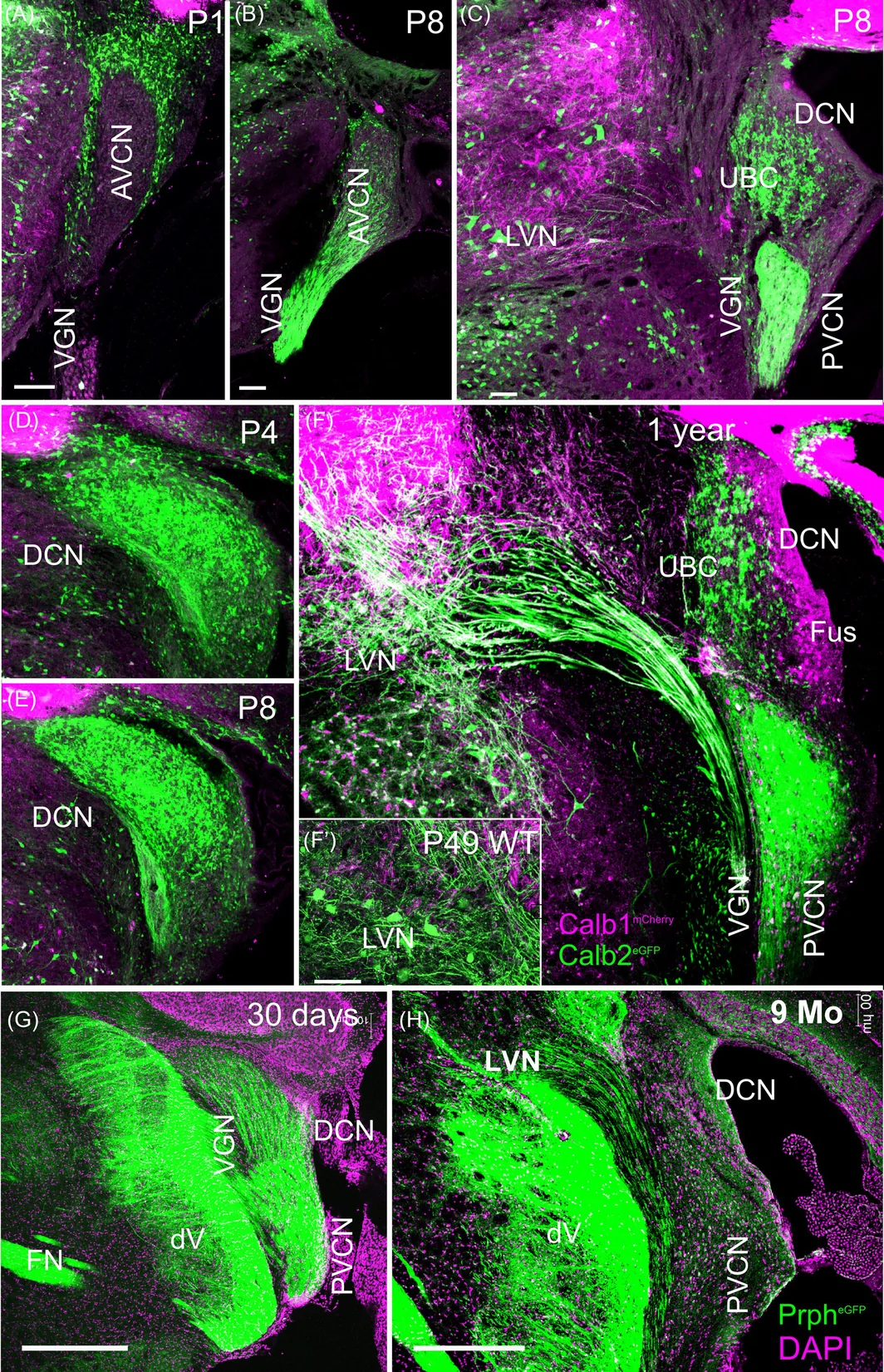
Central projections from VGN and SGN differ in long‐term presence. A significant difference between Calb1 and Calb2 (see F) shows hardly any central projection at P1 (A), while Prph is strongly positive until at least P30 (G). Central projection can be demonstrated by Calb2 to CN via SGN, with Calb2‐positive fibers extending toward the LVN through VGNs (B, C) in P8 old mice. A direct comparison shows a concentration in the central part of DCN that is highly positive for unipolar brush cells (UBC; C, D, E). However, later forming fusiform neurons that are highly positive for Calb1 will become more prominent in older mice (F). The LVN belongs among the largest neurons and can also be labeled with antibody staining for Calb2 (F, F′). Positive Prph is present in the CN and the VGN (G), which later shows positive for the fibers to extend to the LVN, but does not show the central projection. Fus, fusiform neurons; LVN, lateral vestibular neurons; UBC, unipolar brush cells. The bar shows 100 μm.

At first, Calb1 expressions in CN and VN are close to background levels. However, as development progresses, vestibular neurons become positive for Calb1 and expand innervation to the lateral vestibular nuclei (LVN; Figure 9A). Between P4 and P8 and in adults, Calb1‐positivity increases in fusiform (or pyramidal) neurons, which project contralaterally to the inferior colliculi (Figure 9C, F). A small population of neurons between the dorsal cochlear nucleus (DCN) and posteroventral cochlear nucleus (PVCN) becomes highly positive for Calb1. Additionally, neurons near r5 express Calb1 (Figures 9D, E and S5, S6) in contrast to the r4/5 transition region (Figure 9C, F). Note, neurons next to the cerebellum are highly Calb1‐positive Purkinje cells that extend Calb1‐positive neurites into the VN (Figure 9A, C–E), blending with fusiform neurons (Figure 9F). Neurons in the anteroventral cochlear nucleus (AVCN) show Calb1 positivity, with some overlapping with Calb2 expression (Figure S5). Octopus and root cells are Calb1‐positive (Figures S5 and S6), as previously detailed by others.^1^, ^40^, ^62^

#### 3.3.1 Calb2‐positive fibers in both the VN and CN

A change in Calb2 distribution indicates that CN neurons migrate to the area destined to become the AVCN (Figure 9A, B). Similarly, initially widespread Calb2‐positive DCN neurons later mainly localize in granule cells and other neuronal groups, including unipolar brush cells (UBCs). The border between PVCN and DCN features highly Calb2‐positive PVCN neurons, while unipolar brush cells have a different distribution after Calb1‐expressing fusiform neurons (Figure 9C, F). LVN neurons are Calb2‐positive at least during early stages (Figure 9C, F). Note that the Calb2 antibody also labels LVN neurons (insert in Figure 9F′). Conversely, strong Calb2 expression in PVCN fibers appears later, with projections becoming evident around P8 (Figure 9A, B). These fibers intermingle with PVCN neurons (Figure 9C, F), supporting the complex neuronal organization in CN formation.

#### 3.3.2 Prph fibers reach out to VGN and SGN fibers but lose afferent fiber expression to the CN

Prph‐positive VGN fibers are found next to the descending tract of trigeminal fibers (dV) and wedge next to the CN (Figure 9G). The Prph‐positive central projection later loses the innervation from the CN, but extends fibers toward the LVN, compared to the Calb2 expression pattern (Figures 9F, H and S6). A distinction can be made between VGN and SGN fibers: the largest fibers reach out to the VGN (Figure S6), while many small fibers appear from Prph‐positive neurons, reaching nearby VGN and SGN. These fibers are slightly larger compared to Calb2‐expressing SGN fibers. In between are tiny fibers that intersperse Calb1‐positive neurons (Figure S6).

## 4 DISCUSSION

We have shown the dynamic up‐ and downregulation of Calb1‐ and Calb2‐positive HCs, as well as Prph‐positive neurons extending from the auditory and vestibular ganglia to the brainstem nuclei. The relevance of these biomarkers to the two sensory systems is dependent on the developmental stage in the mouse. There is an incomplete overlap of expression that has been documented in other neural systems; for example, the dentate gyrus of the hippocampus.^63^, ^64^ Previous work documented the limited effect of several protein deletions.^11^ Here, we provide a detailed characterization of biomarker expression patterns, which was only possible using knock‐in mouse lines instead of relying on antibody labeling alone.^12^, ^21^, ^42^

### 4.1 The vestibular and cochlear HCs and neurons have different Calb1 and Calb2 expression patterns

We report a unique progression of *Calb1* and *Calb2* in HCs: Cristae HCs start to express Calb1 but shift to Calb2 and increase HCs innervated at the calyx afferents (Figure 1). In contrast, the utricle and saccule are highly positive for Calb2 initially and later develop Calb1‐positive HCs from the striola (Figures 1 and 2). Cochlea IHCs and OHCs overlap with Calb2 expression initially but incompletely segregate into IHCs (Calb2) and OHCs (Calb1) (Figures 3, 4, 5). The largest Calb2‐positive neurons project fibers to innervate the central zone that has the highest Calb1 positivity of canal cristae (Figures 1 and S1). In addition, we document multiple fiber innervation by Prph‐positive neurons (Figures 3 and S1), as described previously.^12^ However, more recent works show a contrasting pattern in adult innervation, in which the calyx and HCs are both positively stained with anti‐Calb2 in the canal cristae.^12^, ^13^, ^15^, ^16^ The expression of Calb1 (mostly) and Calb2 suggests an overlap in the cristae, utricle, and saccule, forming the calyx.^13^

Our findings agree with Dechesne et al.^16^ and show a delayed upregulation of Calb1 expression after P4 (Figure 2). Once upregulated, HCs are still positive for Calb1 in the striola, surrounded by Calb2‐positive HCs (Figure 2).^12^, ^13^, ^19^, ^65^, ^66^ Strong Prph‐positive fibers can be seen throughout these regions (Figure 5), consistent with previous reports (Figure S1).^12^, ^51^

In early development, Calb1 and Calb2 are expressed in both IHC and OHC but become almost exclusive to IHCs with a few remnants in OHCs in the adult (Figures 3, 4 and S2). A different expression pattern occurs at the apex. Initially, only IHCs express Calb2, followed closely by a scattered distribution of Calb1 (Figures 3, 4 and S2). By P4, Calb1 expression declines compared to Calb2 at the tip of the apex (Figures 3 and 4). Calb2‐ and Prph‐neurons appear to form a unique fiber connection, selectively innervating IHCs and OHCs respectively (Figure S2).^14^, ^23^, ^32^, ^67^, ^68^ Additionally, Calb1 is transiently expressed at the GER, before its regression, leading to the formation of the inner sulcus. In summary, we show the longitudinal progression of Calb1 and Calb2 expression in HCs at the cochlear base and apex that differ in the canal cristae and the two maculae, the utricle and saccule.

### 4.2 Vestibular and spiral ganglion neurons are positive for Calb1, Calb2 and Prph

The distribution of VGNs shows a clear division: iVGN innervates PC neurons and a sizable portion of the saccular neurons, while sVGN are connected to the utricle, AC, HC, and a smaller part of the saccule. A sharp boundary split exists between sVGN and iVGN (Figure 7), with some overlap between the two sections of the saccule (Figure S4). In addition, we selectively labeled the vestibulo‐cochlear neurons (VCN)^69^ that originate below the saccule and show an incomplete overlap (Figure 8). Double labeling shows their proximity while revealing distinct fiber segregation between the cochlear apex and the saccule (Figures 8 and S4).

On average, VGN neuron sizes are much larger than those of SGNs (Table 1; Figure 5). Note that the largest neurons are positive for Calb2, while medium‐sized neurons are likely positive for Calb1. In contrast to Calb1, the smallest are the Prph‐positive neurons (Figures 5 and 6). SGNs show a different distribution, with ~8% being Prph, while ~50% of VGNs are positive for Prph (Figures 5 and 6). In contrast, ~40% of both VGNs and SGNs are positive for Calb2, and nearly the same proportion are positive for Calb1 (Figures S2 and S3; Table 1). Overall, our data align closely with previously published analyses, with exceptional differences.^8^

### 4.3 The central projection shows distinct input to the vestibular and cochlear nuclei

Results from analysis using Calb1, Calb2, and Parv antibodies describe the central projection to the CN in the developing rat.^42^ Our current data confirm the findings for Calb1‐ and Calb2‐positive fibers and extend the information for the central projection of Prph‐positive fibers. Specifically, we show that the CN had a ring of neurons at P1 in the AVCN (Figure 8) that were highly positive for Calb1 and Calb2, extending to P4–P8. In the PVCN, we identified a population of octopus and root neurons that are highly positive for Calb2 and/or Calb1, consistent with previous results.^42^ For the DCN, we show a concentration of Calb2‐expressing neurons at P4 that become unipolar brush cells (Figure 9),^70^ as described elsewhere.^1^, ^4^, ^40^, ^71^ In contrast to Calb2, Calb1 shows a unique distribution that is interspersed in the AVCN and PVCN but is highly positive in fusiform neurons (Figure 9). In addition, a small population of Calb1‐positive neurons is found between unipolar brush cells and the PVCN (Figure S5).

Using the parvalbumin antibody, previous work showed that VGN fibers extend to the LVN.^42^, ^72^ We show that Calb2‐positive fibers emanate from VGNs to the LVN and extend further into the medila vestibular nuclei. In addition, Calb1‐positive fibers from the cerebellar nuclei innervate the LVN. Most LVN neurons are positive for Calb2, but a few are positive for Calb1 (Figure S5). SGN fibers lose Prph expression in adults, except for ~5%–8% of neurons that remain positive for Prph.^32^ In contrast, a substantial proportion of VGNs and fibers stay Prph‐positive into adulthood. The largest VGNs are mostly Calb2‐positive, while Prph‐positive neurons are, on average, smaller (Figure S6).

In conclusion, this study highlights the dynamic expression patterns of Calb1, Calb2, and Prph in HCs, VGNs, and SGNs, and their central projections during development and maturation. Key information from the studies includes: (1) Cristae HCs start to express Calb1 but shift to Calb2, and then some become enclosed in calyx afferents. At the same time, there is an incomplete overlap in expression between Calb1 and Calb2. (2) In contrast, the utricle and saccule are highly positive for Calb2 and later develop Calb1‐positive HCs to mostly form a calyx from the striola, supporting distinct separation in Calb2. (3) IHCs and OHCs overlap with Calb2 positivity initially, but incompletely segregate in IHCs, and remain positive for Calb1, which is slightly reduced in IHCs. (4) For spiral and vestibular neurons, most neurons are positive for Calb1 early on, but a significant population turns Calb2‐positive, while a substantial VGN population and type II SGNs are Prph‐positive. (5) Central projections show a delay in Calb1 and Calb2 expression, with intense labeling for Prph fibers. The mature CN and VN are positive for Calb2 at the LVN, while an incomplete segregation of Calb1 and Calb2 is found. Most distinct is the DCN, which is highly positive for Calb2 in unipolar brush cells and segregates from fusiform neurons, which are Calb1‐positive.

### 4.4 Significance

The study underscores the importance of age‐dependent protein expression when using neuronal markers like Calb1, Calb2, and Prph. Our findings offer crucial insights into the temporal regulation of neuronal and hair cell identity and connectivity in the auditory and vestibular systems, which are essential for understanding sensory development, function, and potential repair mechanisms. Further work using knock‐in mice can be used to decide the functional features of the identified labeled cells.

## 5 EXPERIMENTAL PROCEDURES

### 5.1 Mice

To examine peripherin expression over time in the murine auditory system, Peripherin‐eGFP genomic reporter transgenic mice were used.^73^ These Peripherin‐eGFP (*Prph‐eGFP*) mice were backcrossed to the CBA/CaJ background.^74^, ^75^ Polymerase chain reaction (PCR) confirmed genotyping on tail DNA with the following primers: B10Screen5b 5′‐TGCCAGGACCCCACCATTTC‐3′, B10Screen3b 5′‐AGCTGAGACTACAGGCGCGTGCCA‐3′, EGFP‐ProbeR 5′‐GACAACCACTACCTGAGCACCCAGT‐3′.

### 5.2 Calb1‐mCherry and Calb2‐EGFP knock‐in mice generation

Calb1‐mCherry and Calb2‐EGFP mice were generated by Cyagen (Cyagen US Inc). The gRNA targeting the mouse Calb1 gene, the donor vector holding the “2A‐mCherry” cassette, and Cas9 mRNA were co‐injected into fertilized mouse eggs to generate targeted knock‐in offspring. Founder animals were identified by PCR, followed by sequence analysis. These founders were then bred to wild‐type mice to test for germline transmission and F1 animals. PCR screening for F1, PCR primers 1: 5′ arm forward primer: 5′‐CATGTCACTGTGAGGATCTGAATGA‐3′ and 5′KI reverse primer: 5′‐TTTAACAGAGAGAAGTTCGTGGCT‐3′. PCR primers 2: 3′ KI forward primer: 5'‐CCACGAACTTCTCTCTGTTAAAG‐3′ and 3' arm reverse primer: 5′‐TTGTCACCAATAACCAGCAGAGG‐3′. Sequencing was confirmed using those primers. PCR primers 3: 5′ sequence primer: 5′‐TTAATGGACTGGTGTAGCAAGCA‐3′ and PCR primers 4: 3′ sequence primer: 5′ CACTACGACGCTGAGGTCAAGAC‐3′. Once confirmed, the heterozygotes were mated to obtain homozygotes. PCR confirmed genotyping from tail DNA with the following primers: Primers 5: 5′‐TTAATGGACTGGTGTAGCAAGCA‐3′. Primers 6: 5′‐GCTTTAACAGAGAGAAGTTCGTGG‐3′. Primers 7: 5′‐GTACTGACTGGCCTAAGCATGGTC‐3′. Homozygotes: 250 bp/1225 bp and wild‐type allele: 451 bp.

The gRNA targeting the mouse Calb2 gene, along with the donor vector containing the “2A‐EGFP” cassette and Cas9 mRNA, was co‐injected into fertilized mouse eggs to generate targeted knock‐in offspring. F0 founder animals were identified by PCR, followed by sequence analysis, and bred with wild‐type mice to test for germline transmission and produce the F1 generation. Similarly, the gRNA targeting the mouse Calb1 gene, the donor vector with the “2A‐mCherry” cassette, and Cas9 mRNA were co‐injected into fertilized mouse eggs to create targeted knock‐in offspring. F0 founder animals were identified by PCR and sequence analysis, then bred with wild‐type mice to test germline transmission and produce F1 offspring. The mouse lines were viable, with body weight and lifespan comparable to heterozygous and wild‐type littermates (~2–3 years). The expression of EGFP in Calb2‐positive and mCherry in Calb1‐positive cells was validated using single‐molecule fluorescence in situ hybridization (smFISH) and Calb2‐ and Calb1‐specific antibodies. The peripherin‐eGFP transgenic mouse line is described.^32^, ^73^ Additionally, the Calb1 and Cab2 mouse lines and reporter‐specific expression has been described elsewhere.^63^, ^76^

Founder animals were identified by PCR, followed by sequence analysis. These founders then bred to wild‐type mice to evaluate germline transmission and F1 animals. PCR screening for F1, PCR primers 1: 5′ arm forward primer: 5′‐ATCACTTGAGGTTGGTGAAGTCTG‐3′ and 5′ KI reverse primer: 5′‐TTAACAGAGAGAAGTTCGTGGCTC‐3′. PCR primers 2: 3′ KI forward primer: 5′‐GACGTAAACGGCCACAAGTT‐3′ and 3′ arm reverse primer: 5′‐TACTCATCTTCAGTTGCCCTGAC‐3′. The sequencing was confirmed with the following PCR primers: 3′ 5′ sequence primer: 5′‐AGAAGTCGTGCTGCTTCATGTGGTC‐3′ and 3′ sequence primer: 5′‐GCCACAACGTCTATATCATGGC‐3′. Once confirmed, the heterozygotes were mated to obtain homozygotes. PCR confirmed genotyping on tail DNA with the following primers: Primers 4: 5′‐AGTTGATAGGAAGGTCCATTCGC‐3′, 5′‐TCCACTCTGTGGGACTCAGAAG‐3′, band size: 581 bp for homozygotes. Primers 5: 5′‐GCCACAACGTCTATATCATGGC‐3′ and 5′‐TCCACTCTGTGGGACTCAGAAG‐3′, product size: 542 bp for wild‐type allele.

All experiments were performed according to the United States Animal Welfare Act and the National Institutes of Health's policy to ensure proper care and use of laboratory animals for research, and under established guidelines, supervision, and approved protocols by the Institutional Animal Care and Use Committee (IACUC:2024‐1202) of the University of Phoenix, Arizona.

### 5.3 Fixation and tissue preparation

Mice were anesthetized and intracardiac perfused at various stages (E18.5, P1, P4, P8, P14, P28, P46, and 1 year) with 4% paraformaldehyde in paraformaldehyde (PFA) (pH 7.6) with 0.3 M sucrose to keep neuronal structural integrity. The head was removed and shipped in 0.4% PFA with 0.3 M sucrose on ice, protected from light. The head was then bisected, and the brain halves and temporal bones were removed. The sample was sectioned coronally at a thickness of 50–90 μm using a comprestome. To view, the slides were washed in phosphate buffered saline (PBS) for approximately 4 min to remove the embedding medium, and the coverslips were mounted using Fluoromount‐G with DAPI (Thermo Fisher Scientific; #00‐4959‐52). Care was taken to protect the samples from light at all stages of the procedure.

The temporal bones older than P8 were decalcified in a 0.25 M ethylenediaminetetraacetic acid solution (research products international (RPI); # E57020) for up to 5 days, with daily solution changes protected from light. Decalcified cochleae were washed in PBS, micro‐dissected, and the tectorial membrane was removed. Cochlear turns were flat‐mounted in glycerol or Fluoromount‐G with 4′,6‐diamidino‐2‐phenylindole (DAPI) for viewing.

### 5.4 Immunofluorescence

We used antibodies to confirm the transgenic data. Whole‐mount, dissected cochleae were blocked and permeabilized with 30% goat serum in 1× PBS + 0.3% Triton X‐100 for 24 h, then incubated in primary antibody solution 1× PBS + 0.1% Triton X‐100 + antibodies for 24–48 h at 4°C. The primary antibodies used were here:

**Table:** 

Antibodies	Target antigens	Host organism	Manufactor	RRID	Dilution
Anti‐Myo7a	Rat, mouse	Rabbit	Proteintech	RRID:AB_1 0734441	1:500
	Polyclonal		Cat# 20720‐1‐AP		
Anti‐acetylated tubulin	Mouse polyclonal	Mouse	Sigma‐Aldrich	RRID:AB_609894	1:500
			Cat#T7451		

Abbreviations: RRID, Research Resource Identifiers.

After several PBS washes (3 × 1 h) at room temperature, the samples were incubated in species‐specific secondary antibody solution at 4°C for 12–24 h. The secondary antibodies used were as follows:

**Table:** 

Antibodies	Manufacturer	RRID	Dilution
Goat anti‐Mouse IgG, Alexa Fluor 488	Invitrogen	A‐28175 RRID:AB_253616	1:500
Goat anti‐Rabbit IgG, Alexa Fluor 546	Invitrogen	A‐11010	1:500
		RRID:AB_2534077	

Some samples used DAPI (Sigma‐Aldrich D9542, 1 μg/mL) as a nuclear counterstain. Finally, the samples were washed several times (3 × 1 h) in PBS before viewing, using glycerol as mounting media and coverslips.

### 5.5 Tracing with NeuroVue

We described in detail the use of neuron tracing with NeuroVue, maroon, red, and jade.^77^ We selectively labeled the utricle, AC, and HC in NeuroVue Jade, using red for the saccule and maroon for the PC. In addition, we labeled the vestibular ganglia from the apical tip with maroon, combined with cerebellum insertion with red. We used jade in the inner ear efferent by inserting it into the middle of r4 (IEE). The whole‐mount preparation was used to visualize neuronal fiber tracing and the innervation of the IEE fibers.

Images were obtained using a Leica SP8 scanning laser or a Zeiss 700 confocal microscope, analyzed with Leica LAS X or Zen 3.8 software, and processed with CorelDRAW Graphics Suite. Images were taken at 1–10 μm thick optical sections to compile into a given stack in up to four different colors (405, 488, 552, and 638 nm laser lines) using three different magnifications for each confocal system (10× with a 0.6 NA; 20× with a 0.95 NA; 63× with a 1.4 NA for the Leica, 10× with 0.5 NA; 20× with 0.8 NA; 40× with 1.4 NA for the Zeiss).

### 5.6 Quantification

For the analysis of VGNs and SGNs, we used mice at E18.5, P1, P2, P4, P8, P28, and P46. The last two stages involved adult mice, with at least four control and four knock‐in mice. Optical sections were performed using a confocal microscope with 40× imaging to measure the largest diameter (in μm) and area (in μm^2^) of labeled neurons. The distribution followed the size difference distribution of Calb1, Calb2, and Prph alone or in combination, including DAPI‐stained material. Tables are provided for size variations, and the largest diameter is measured using confocal images. We used a HALO system to measure the exact sizes of distinct neurons, focusing on finding the best size. Using HALO for the analysis of various brain sections requires all images to be converted into TIFF files and downloaded into the system. In quantifying these images, the first layer found the diameter of the neurons, and the second layer found the area of the same cell. The system then compiles the data into separate tables, organized by layer. The tables were transposed into an Excel spreadsheet where the mean intensities were averaged, and the standard error was calculated using Microsoft Excel. Statistical significance was performed with one‐way analysis of variance.

## CONFLICT OF INTEREST STATEMENT

None of the authors has a conflict of interest to disclose.

## Supporting information


**Figure S1.** *Calb1* and *Calb2* have unique hair cell distribution. (A, B–B″) shows that they have fibers that reach out to the AC, HC, and PC to innervate the Calb2 positive and largest cells. In contrast, Prph shows many thinner fibers that reach out to the HC (C) and U (D). This does not reach out to the Calb1 striola region. The bar indicates 100 μm.
**Figure S2.** Cochlear hair cells show a mix of Calb1 and Calb2. (A–C″) positive show the expression for IHCs and OHCs. Note that the OHC is closer to the apical tip and has a few IHC neurons with gaps (*) and most tip IHCs (A, B). OHC is most positive for Calb1, while Calb2 is most positive for IHCs (C–C″). Calb1 and Prph have a unique distribution that forms the most lateral to innervate the radial fibers, while the more medial modiolus is positive for Calb1 (D). A single preparation that shows the cochlear hair cells, the spiral ganglion neurons (SGN), and the fibers to form the modiolus (E). In addition, we have the inferior vestibular ganglion neurons (iVGN) to reach out to the PC (to PC) and smaller neurons that reach out to the saccule (to Sac). The bar indicates 100 μm.
**Figure S3.** Two whole mount images show the distribution of Calb1, Calb2, and Prph. (A–C) Note that fibers are thick, and few are positive for Calb2, next to the VGN (C). According to Fernandez et al. (1990), the Calb2‐positive fibers/neurons are calyx‐only and makeup only 10% of all VG neurons. In contrast, many smaller Prph neurons are embedded with Calb1 (D), which shows many fibers leaving the VGN. The Prph‐positive fibers/neurons are bouton‐only and makeup about 20%, about twice as many as the Calb2. The bar indicates 100 μm.
**Figure S4.** A vestibulo‐cochlear neuron (VCN) forms next to the cochlear nerves (CN). (A, A′, B) Shows that forming a separate, more ventral branch parallel to the saccule. Note that iVGN and sVGN come from two branches to provide saccular neurons (A, B). Higher power shows VCN next to the saccule neurons that intermingle (A″, B′). The bar indicates 100 μm.
**Figure S5.** Cochlear neurons are positive for Calb1 and Calb2. (A) shows innervation from the AVCN (A) to the DCN and a unique UBC and Fus neurons (B) distribution. VGN fibers reach out to innervate the LVN (C, D), which shows a unique distribution of Calb1 positive (*) with fewer VGN fibers, while the neurons positive for Calb2 show more terminal surrounding the LVN neurons (&). A positive Prph shows the descending tract of the trigeminal (dV) next to the many fibers of the VGN that also send fibers to innervate the cochlear neurons with SGN (C, D). The bar indicates 100 μm.
**Figure S6.** A distinct fiber of VGN and SGN shows small and extensive fibers, respectively. (A) SGN's thinner fibers reach the CN (A, A'). Note the root cells are particularly innervated with punctate SGN fibers (A″). In contrast to the Calb2 fibers, Prph fibers have many that can reach the VGN and SGN fibers. The bar indicates 100 μm.
