# Supplementary material for: The segregation of Calb1, Calb2, and Prph neurons reveals distinct and mixed neuronal populations and projections to hair cells in the inner ear and central nuclei
Source: Dev Dyn. 2025 Oct 30;255(9):845–66. doi: 10.1002/dvdy.70093 (PMC12907763; doi:10.1002/dvdy.70093)
Supplement: Supplementary file 1 [file DVDY-255-845-s001.docx]

Supplement Fig. 1. *Calb1 and Calb2 have unique hair cell distribution.* (A, B-B”) shows that they have fibers that reach out to the AC, HC, and PC to innervate the Calb2 positive and largest cells. In contrast, Prph shows many thinner fibers that reach out to the HC (C) and U (D). This does not reach out to the Calb1 striola region. The bar indicates 100 µm.

Supplement Fig. 2. *Cochlear hair cells show a mix of Calb1 and Calb2.* (A-C”) positive show the expression for IHCs and OHCs. Note that the OHC is closer to the apical tip and has a few IHC neurons with gaps (*) and most tip IHCs (A, B). OHC is most positive for Calb1, while Calb2 is most positive for IHCs (C-C”). Calb1 and Prph have a unique distribution that forms the most lateral to innervate the radial fibers, while the more medial modiolus is positive for Calb1 (D). A single preparation that shows the cochlear hair cells, the spiral ganglion neurons (SGN), and the fibers to form the modiolus (E). In addition, we have the inferior vestibular ganglion neurons (iVGN) to reach out to the PC (to PC) and smaller neurons that reach out to the saccule (to Sac). The bar indicates 100 µm.

Supplement Fig. 3. *Two whole mount images show the distribution of Calb1, Calb2, and Prph.* (A-C) Note that fibers are thick, and few are positive for Calb2, next to the VGN (C). According to Fernandez et al. (1990), the Calb2-positive fibers/neurons are calyx-only and makeup only 10% of all VG neurons. In contrast, many smaller Prph neurons are embedded with Calb1 (D), which shows many fibers leaving the VGN. The Prph-positive fibers/neurons are bouton-only and makeup about 20%, about twice as many as the Calb2. The bar indicates 100 µm.

Supplement Fig. 4. *A vestibulo-cochlear neuron (VCN) forms next to the cochlear nerves (CN).*  (A, A’, B) Shows that forming a separate, more ventral branch parallel to the saccule. Note that iVGN and sVGN come from two branches to provide saccular neurons (A, B). Higher power shows VCN next to the saccule neurons that intermingle (A”. B’). The bar indicates 100 µm.

Supplement Fig. 5. *Cochlear neurons are positive for Calb1 and Calb2*. (A) shows innervation from the AVCN (A) to the DCN and a unique UBC and Fus neurons (B) distribution. VGN fibers reach out to innervate the LVN (C, D), which shows a unique distribution of Calb1 positive (*) with fewer VGN fibers, while the neurons positive for Calb2 show more terminal surrounding the LVN neurons (&). A positive Prph shows the descending tract of the trigeminal (dV) next to the many fibers of the VGN that also send fibers to innervate the cochlear neurons with SGN (C, D). The bar indicates 100 µm.

Supplement Fig. 6*. A distinct fiber of VGN and SGN shows small and extensive fibers, respectively.* (A) SGN’s thinner fibers reach the CN (A, A’). Note the root cells are particularly innervated with punctate SGN fibers (A”). In contrast to the Calb2 fibers, Prph fibers have many that can reach the VGN and SGN fibers. The bar indicates 100 µm.
